# Valorization and Utilization of Food Wastes and By-Products: Recent Trends, Innovative Technologies and Sustainability Challenges

**DOI:** 10.3390/foods13010009

**Published:** 2023-12-19

**Authors:** Noelia Betoret, Ester Betoret, Virginia Teresa Glicerina

**Affiliations:** 1 Instituto Universitario de Ingeniería de Alimento—FoodUPV, Universitat Politècnica de València, 46022 València, Spain; 2 Department of Agriculture, Forest and Food Sciences, University of Turin, 10095 Grugliasco, Italy; viginiateresa.glicerina@unito.it

## 1. Introduction

The recovery of food by-products and waste is an issue of universal concern, as every year the food industry generates a huge amount of waste and by-products from a variety of sources. Food waste has a significant impact on food and nutrition security, food quality and safety, and environmental protection. Due to the large quantities of organic matter generated in a short period of time, these products represent a critical sustainability issue. The gradual depletion of natural resources and the increasing demand for food have led to the need to limit energy consumption, minimize costs and reduce waste by developing strategies for the recovery and utilization of food waste and by-products [1]. In fact, in most cases, these products have a high potential for recycling that can be achieved through the recovery of their bioactive compounds, plant protein extraction, transformation into powdered ingredients, or controlled fermentation. All of these actions will contribute to solve the challenges of climate change and food system sustainability, creating new opportunities and benefits for all stakeholders.

## 2. Innovative Technologies

As food waste contains significant amounts of valuable components, several action plans for the evaluation and utilization of wastes and by-products have been developed by various countries worldwide. The scope of these action plans is aimed at evaluating and valorizing agricultural and food waste through physical–chemical (extraction, hydrolysis, dehydration, isomeration, etc.), biological (enzymatic hydrolysis, fermentation, anaerobic degradation, etc.) or thermochemical (direct combustion, pyrolysis, etc.) processes. In recent years, “innovative” technologies with important applications in food processing have been used to improve the extraction of valuable components, increase yields and improve the quality and functionality of extracts. These include electro-technologies (pulsed electric fields, high-voltage electrical discharges, and non-pulsed electric fields, e.g., ohmic heating and moderate electric field); laser ablation; ultrasound-assisted extraction (UAE); radiofrequency drying; high hydrostatic pressure (HHP) and pressurized fluids (sub- and supercritical fluid extraction); and nanotechnology, among others. Electric pulses induce the size reduction and disruption of cell membranes, facilitating the recovery of natural food pigments and bioactive components such as lycopene, polyphenols, carotenoids, etc. [2], from plant product residues using eco-friendly solvents. Electrical pulses have been applied to winemaking-derived by-products and fruit bagasse [3]. Moreover, ohmic-assisted hydro-distillation has been used to improve thermal efficiency in order to reduce the processing time and operational cost in the extraction of essential oils from aromatic plant residues [4,5]. Laser ablation has been used as a means to enhance the heat and mass transfer processes. It has been applied to the extraction of macromolecular components from plant sources. The extraction of pectin and aromatic compounds from edible matrices has shown promising results [6]. Ultrasound technology uses cavitation to break up cellular tissue. This allows the solvent to penetrate deeper into the raw material. The high hydrostatic pressure (HHP) approach involves subjecting materials to pressures reaching up to 1000 MPa. HHP increases plant cell permeability, increasing components’ diffusivity. Furthermore, the solubility of compounds is greater when the pressure increases. Ultrasound and HHP have been combined for pectin and polyphenol recovery from tomato peel waste [7]. The use of pressurised fluids, such as supercritical and sub-critical fluid extraction, as well as gas-assisted mechanical expression, has garnered attention from both academic researchers and industry professionals. According to Wijngaard et al. [8], pressurized liquid extraction and supercritical fluid extraction are extraction techniques with the ability to increase target molecule specificity and reduce waste solvent production. Supercritical CO_2_ extraction has been applied for the extraction of hydrophobic compounds, such as carotenoids from tomato peels [9]. However, despite these promising results, constant efforts and innovation are needed to achieve sustainable food production.

## 3. Recent Trends and New Challenges

Waste and by-product reduction will be one of the key challenges in the coming years to achieve the objectives of the 2030 Sustainable Development Goals, as defined by the United Nations (UN) [10]. In addition, the “Waste Framework Directive” (2008) focused on additional targets of waste and by-product reduction, with the specific goals of reductions of 55% by 2025, 60% by 2030 and 65% by 2035 [11].

One of the most recent trends in the use of food by-products and waste is the interest in the production of alternatives to animal protein. The solid-state fermentation of crops and their by-products is being investigated to obtain protein resources. Solid-state fermentation promotes the conversion of waste biomass into edible ingredients or enzymes, reducing the environmental impact of the food industry. The fermentation substrate is diversifying from the current mainly agro-industrial waste; for example, olive cake, maize stalk and grape stalk have been investigated to produce plant-based protein for use as fermentation substrates [12]. The increase in the protein content of these crop by-products after solid fermentation was quantified as 25, 126 and 77%, respectively [12]. These solutions can accelerate the decoupling of the food industry from arable land and enable the production of high-value-added crops.

Although considerable efforts have been made, there are still many challenges to overcome. More research is needed to identify solutions to reduce food waste at the different stages of the food supply chain. This Special Issue aims to focus on the new trends and innovative technologies that may be applied to valorize and re-use food waste by-products, with the ultimate goal of increasing food and environmental sustainability.
